# Measuring Power of Earth Disturbances Using Radio Wave Phase Imager

**DOI:** 10.3390/jimaging9100228

**Published:** 2023-10-20

**Authors:** Radwan N. K. Sharif, Rodney A. Herring

**Affiliations:** 1 Department of Mechanical Engineering, MENG, University of Victoria, Victoria, BC V8W 2Y2, Canada; 2 Centre for Advanced Materials and Related Technology (CAMTEC), Department of Mechanical Engineering, MENG, University of Victoria, Victoria, BC V8W 2Y2, Canada; rherring@uvic.ca

**Keywords:** software-defined radio, radio wave imager, ionospheric waves, phase imaging method with wavevectors, power stations, transmission line losses

## Abstract

Numerous studies have investigated ionospheric waves, also known as ionospheric disturbances. These disturbances exhibit complex wave patterns similar to those produced by solar, geomagnetic, and meteorological disturbances and human activities within the Earth’s atmosphere. The radio wave phase imager described herein measures the power of the ionospheric waves using their phase shift seen in phase images produced by the Long Wavelength Array (LWA) at the New Mexico Observatory, a high-resolution radio camera. Software-defined radio (SDR) was used for processing the data to produce an amplitude image and phase image. The phase image revealed the ionospheric waves, whereas the amplitude image could not see them. From the phase image produced from the carrier wave received at the LWA, the properties of the ionospheric waves have been previously characterized in terms of their energy and wave vector. In this study, their power was measured directly from the phase shift of the strongest set of ionospheric waves. The power of these waves, which originated at Albuquerque, the local major power consumer, was 15.3 W, producing a power density of 0.018 W/m^2^. The calculated power density that should be generated from the local power generating stations around Albuquerque was also 0.018 W/m^2^, in agreement with the experimentally measured value. This correspondence shows that the power generated by power stations and being consumed is not lost but captured by the ionosphere.

## 1. Introduction—Earth’s Ionosphere Response to Waves

Changes in the ionosphere, the uppermost part of the Earth’s atmosphere, are known as ionospheric disturbances. These disturbances, often referred to as ionospheric waves, can be triggered by a diverse range of natural phenomena, including solar flares, geomagnetic storms, meteor impacts, lightning, volcanic eruptions, and earthquakes, as well as man-made disturbances, such as explosions and electricity generated and transported of power stations [1,2,3,4]. Traveling ionospheric disturbances (TIDs) are generated by atmospheric waves that travel across the ionosphere region. Scientists have employed various techniques to identify and analyze these disturbances to better comprehend the mechanics of wave propagation and the geophysical causes of TIDs. Therefore, TIDs have been detected for years using ground-based observations, namely, radio techniques, such as ionosondes, incoherent scatter radars, and High-Frequency (HF) Doppler sounders [5,6,7,8,9]. More recently, GPS total electron content (TEC) has been used to create 2D images of TIDs in places like Japan [10,11] and the United States [12]. Understanding TIDs is critical to improving our ability to anticipate and mitigate their impact on our technology and environment.

TIDs are disruptions in the number of electrons in the ionosphere caused by a disturbance in atmospheric gravity waves (AGWs). These waves collide with neutral particles traveling through the thermosphere and ionosphere. The resulting movement of the neutral gas sets the ionosphere in motion, causing variations in electron density.

The characteristics of TIDs are influenced by factors such as their origin, the conditions of their spread, damping, and the extent to which they are reduced over time. Therefore, predicting TID periods, amplitudes, speeds, and directions at a given location remains challenging because of the complex nature of TIDs and the lack of comprehensive observational data [13].

Several articles have mentioned techniques for identifying traveling ionospheric TIDs [14,15,16,17,18]; still, additional observations are needed to fully understand the physics of wave propagation and geophysical source phenomenology. However, in more detail, these articles study physical events, such as the tsunami associated with the Tohoku earthquake that occurred on 11 March 2011, which was examined using data on total electron content (TEC) from around 4000 GPS devices dispersed across the continental United States. This work proved that the accompanying TIDs were detected during the tsunami, which traveled across the Pacific Ocean to Japan’s west coast for several hours. The researchers computed different TID properties, including horizontal wavelength, velocity, and duration. A two-dimensional representation of TEC disturbances was made possible using this network of GPS receivers [19].

The characteristics and behavior of ionospheric waves have been the subject of research. For instance, Hunsucker and Hargreaves thoroughly explain ionospheric waves, including how they are created, spread, and affect the ionosphere. They reviewed several ionospheric wave types, such as gravity, acoustic, and plasma waves, and explained how they interact with the ionosphere [20]. 

AGWs are atmospheric waves that occur when air density and pressure are disturbed by wind, heat, or terrain. When gravity restores equilibrium, the waves propagate to bring the atmosphere back to balance after it has been disrupted by the external force. These waves, generally described as buoyancy waves, may significantly alter the weather and environment [21,22,23].

Other natural disasters, including earthquakes, have generated waves affecting the ionosphere. In 2016, the authors of [24] conducted a study on the Nepal earthquake of 25 April 2015, using GPS technology to measure changes in the ionosphere. They found that the earthquake caused seismic-traveling ionospheric disturbances (STIDs) propagating at 2.4 km/s horizontally.

Another study by Harrison observed a correlation between the responses of the lower ionosphere and seismic activity in the region prior to an earthquake [25].

The generation of waves on the ionosphere and magnetosphere by power line emission (PLE) and power line harmonic radiation (PLHR) is a subject of growing concern due to their potentially harmful effects [18,26,27,28,29,30]. 

In more recent experimental investigations, ionosphere wave sources have been traced back to the vicinity of local power generating stations [31]. The primary site of power consumption was identified as Albuquerque, New Mexico. The success of this endeavor could be attributed to the novel measurement technique employed, which used a wavevector analysis of the ionosphere waves detected at two distinct locations, LWA1 and LWA-SV.

The study reported here introduces a novel approach for measuring the power of waves in the ionosphere and investigates their frequency characteristics, representing the first instance of such measurements. This report involves using a terrestrial radio-wave transmitter to illuminate the ionosphere and measure the power of its ionospheric waves. The radio waves were transmitted from the ground and reflected back to a two-dimensional array of passive receivers/antennas, allowing for the relative phase imaging of the ionosphere waves and revealing their temporal and spatial evolution. This study used Fourier analysis to determine the power, energy, and location of the ionosphere wave generation based on amplitude, frequency, and wavevectors. Furthermore, this study explored the possibility of a correlation between ionosphere waves and local Earth disturbances, such as power generating stations.

### Response of the Ionospheric System to Man-Made Disturbances

The impact of power line emission (PLE) and power line harmonic radiation (PLHR) on the ionosphere and magnetosphere have been a growing concern. Since the 1970s, many publications have reported on PLHR and PLE monitoring on the ionosphere PLHR is a type of electromagnetic wave at harmonic frequencies of 50 or 60 Hz, observed in the ionosphere (radiated) and caused by electric power systems on Earth’s surface. PLE refers to a form of emission that occurs at frequencies of 50 or 60 Hz. Because their frequencies are in good accord with comparable ground power system frequencies, PLHR and PLE are thought to be connected to ground electric power systems [10,11]. Other investigations have shown that increased electron precipitation and changes in the ionosphere and magnetospheric currents may result from the penetration of PLHR into the lower ionosphere, potentially impacting thunderstorm activity [27,28,29,30]. Some of the artificial disturbances in the ionosphere were PLE and PLHR. These articles considered PLHR as a type of pollution originating from the power system [32,33]. In 1975, The pioneering work of [34,35] revealed that very low–frequency (VLF) line radiation existing in the Earth’s magnetosphere has a frequency close to 60 Hz. It was proposed that harmonic radiation from the Canadian power grid penetrates a whistler duct in the magnetosphere. Luette et al. in 1977 showed that PLHR had the ability to stimulate the strongest waves on the magnetosphere [36]. In 1979, Park and Miller studied magnetospheric wave intensity between 2 to 4 kHz [37]. They identified that the chorus (discrete narrowband emissions) activity indicated a noticeable minimum on Sundays compared to the rest of the week because of low electrical power usage. Other researchers, specifically Matthews and Yearby in 1981 [35], found that the characteristics of VLF line radiation of magnetospheric waves detected at one location, Halley (75.5674° S, 25.5165° W) British Overseas Territory), were usually equivalent to the PLHR observed at another location, Siple (75.916667° S, 83.916667° W). The analysis of these events employing the time–frequency spectrogram of the electric field strength revealed numerous parallel horizontal spectral lines separated by 50 Hz/100 Hz or 60 Hz/120 Hz, equivalent to the local terrestrial power system’s working frequency. Another study [38] showed that broadcast transmitters, electric power plants, and their associated power line transmission losses, as well as electric power losses in heavy industries, are all potential sources of ionosphere disturbances that may generate ionosphere waves. 

In other research, the characteristics and connections between the two types of radiation were investigated using DEMETER satellite data to identify PLHR and PLE events in the ionosphere above China [26]. The electric field power density time–frequency spectrograms displaced 133 PLHR events with the 50 Hz spectral line frequency. They were very closely connected to the frequency of the ground power grid [26]. 

More recent experiments found the sources of the ionosphere waves to be local power generating stations, and the primary place of power consumption was Albuquerque, NM. This capability was enabled by the measurement of the wavevectors of the ionosphere waves made possible by the detection of the waves from two locations, LWA1 and LWA-SV [31]. 

The study reported here involves illuminating the ionosphere with radio waves from a terrestrial radio-wave transmitter and builds upon the previously reported capability of using this device to measure the ionosphere waves’ wavevectors. The radio waves terrestrially transmitted were reflected back from the ionosphere to the ground and were captured by a two-dimensional array of passive receivers/antennae. From the time-tagged received signals at each receiver, the ionosphere waves could be relative phase imaged, revealing their spatial and temporal evolution. From a Fourier analysis of the ionosphere waves’ phase image, which contained sets of phase shifts, frequencies, and wavevectors, properties of the strongest set of waves, such as the power, energy, and location of generation of the ionosphere waves, were determined. A possible relationship between the waves of the ionization layer and man-made local Earth disturbances, namely local power generating stations, is suggested by matching the measured power to the calculated power.

## 2. Materials and Methods

### 2.1. SDR Experimental Method

Through the transmission of radio signals almost vertically from the Earth’s surface, the SDR Earth Imager effectively acquires significant data from the ionosphere. Following their interaction with the ionosphere, these signals make their way back to the Earth’s surface. Throughout this involved procedure, the antenna array camera diligently captures and records these signals, enabling the creation of amplitude and phase imager. Of particular interest, the phase imagery reveals the presence of a phase shift, represented as Δ*φ*, which indicates the variation in the path length (L) that the radio wave has traversed.

Δ*φ* = 2*π* (L/λ)= 2*π* f (L/c) (1)

where c represents the speed of light, λ denotes the wavelength, and the f represents the frequency of the carrier wave. The phase shift indicates the disparity in the path length covered by the radio wave traveling from the transmitter to the antenna array detector. This phase shift holds significance as it aids in assessing the strength or power of the ionospheric wave.

Data were collected at two distinct locations in New Mexico, LWA-1 and LWA-SV. One dataset was collected at the Long Wavelength Array (LWA)-SV station at the Sevilleta National Wildlife Refuge in central New Mexico [39]. The other set of data was obtained using the LWA-1 radio telescope array, which is positioned in northern New Mexico and has a diameter of 100 m. This radio telescope array is associated with the University Radio Observatory [40].

#### 2.1.1. Transmitter and Dataset Collection

The transmitter was placed in Santa Fe and transmitted radio waves with frequencies of 5.3570 MHz on 8 January 2021, as shown in Table 1.

#### 2.1.2. Receivers and Transmitter Distances

**Figure 1:**
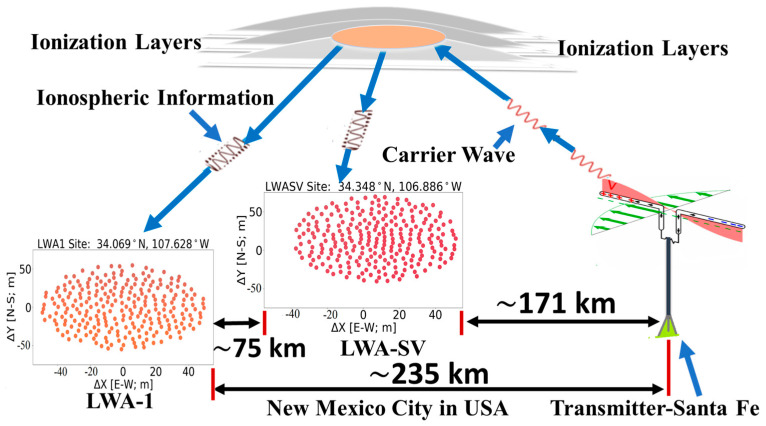
The distance between the transmitter of the carrier wave in Santa Fe and the two receiver arrays at the LWA stations, LWA1 and LWA-SV, as well as the distance between the two stations.

LWA-SV and the Santa Fe transmitter are separated by around 171 and 235 km, respectively, from the LWA-1 station. Figure 1 visually represents the approximate 75 km between the two stations, LWA1 and LWA-SV [40].

#### 2.1.3. LWA-1 and LWA-SV Experimental Approach 

The LWA Software Library (LSL) was built to manage the LWA-1 and LWA-SV data formats and make them accessible to basic analytic tools [41]. The carrier wave was extracted from the frequency range employing a short-time Fourier transform (STFT). For the LWA-SV and LWA-1 data, the relative unwrapped phase difference of the antenna reference of this channel was computed.

#### 2.1.4. Amplitude and Phase Images from the Antenna Array Receiver

**Figure 2:**
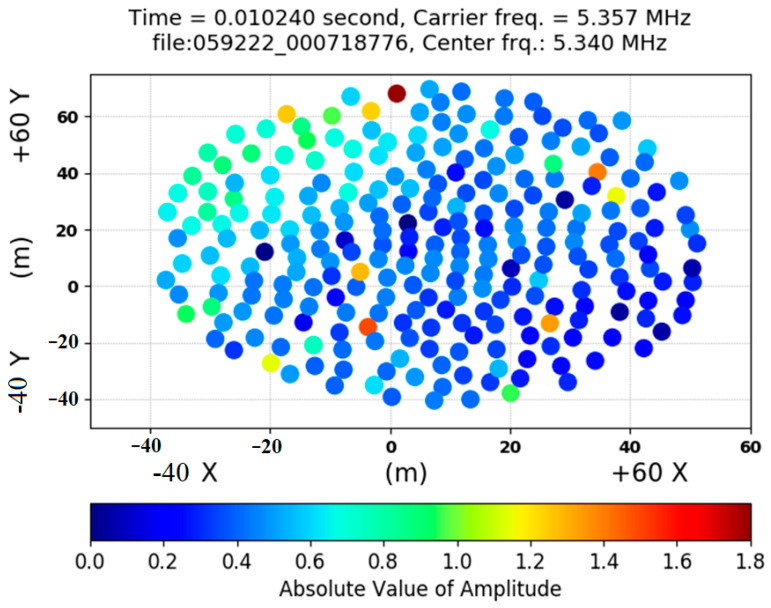
An amplitude image produced from the amplitude signals received at each antenna location.

**Figure 3:**
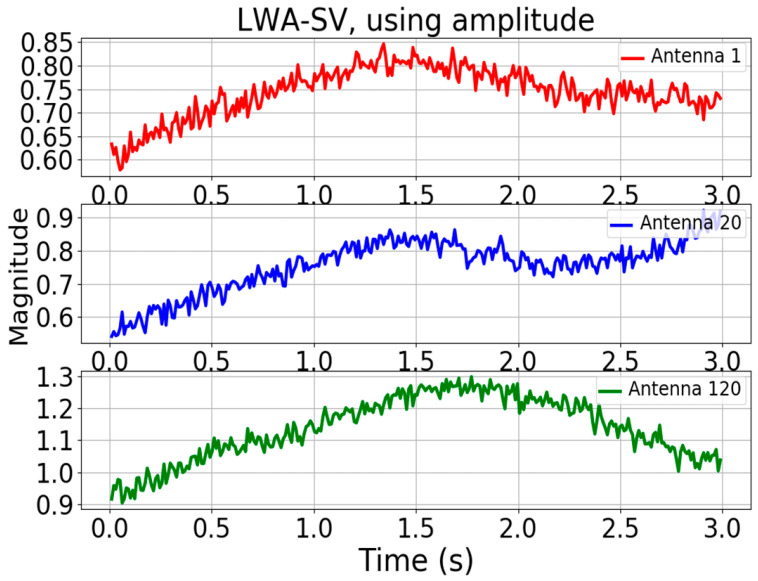
Three plots displaying the amplitude signals received versus time over a 3 s period at three antennas, respectively.

**Figure 4:**
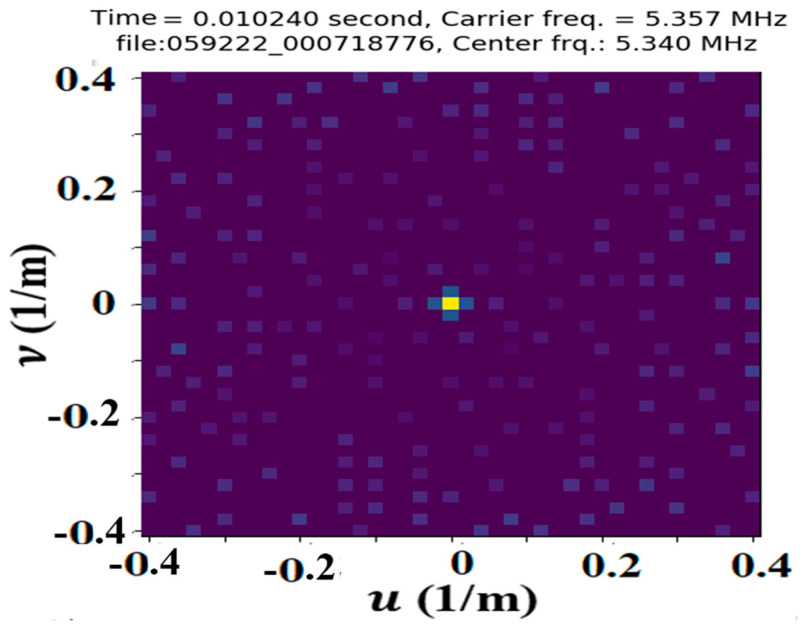
The Fourier image of the amplitude image in Figure 2, which does not reveal any strong set of waves.

An amplitude image produced from the carrier radio wave does not display any wave information, as shown in Figure 2, Figure 3 and Figure 4. The plot in Figure 2 shows the absolute values of real numbers and the amplitude image obtained from performing spectrogram analysis on the raw antenna data. Specifically, the plot is based on selecting the transmitted carrier bin from 256 antennas.

The phase difference between the signals received at each individual antenna in the array is the phase shift. Since each antenna contributes one phase measurement, the antenna array works as a camera to produce a phase image, which reveals the waves within the ionosphere. The phase shift is directly proportional to the amplitude of the ionospheric waves. 

### 2.2. Spatial Phase Image

**Figure 5:**
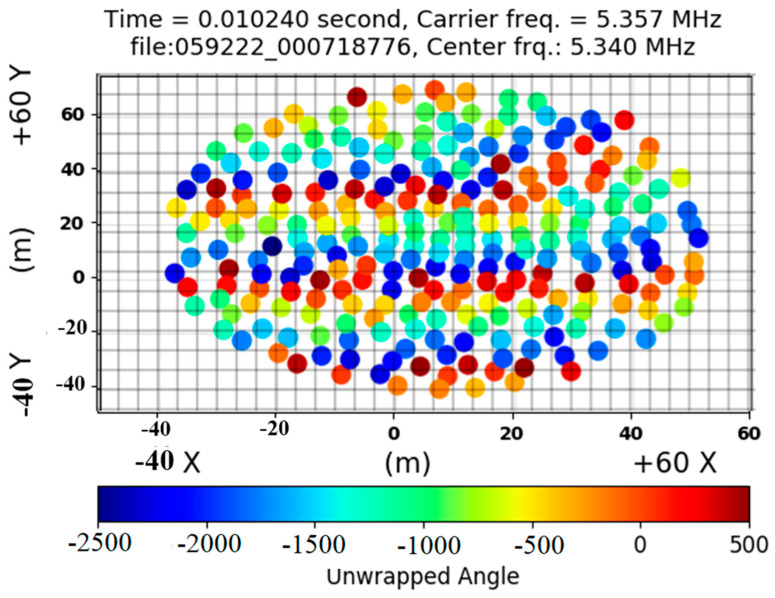
The grid showing antenna locations is divided into cells containing relative unwrapped phases to yield the phase image. A dominant vertically oriented wave is obvious.

The antenna placements in the x- and y-directions are depicted in Figure 5. By using unwrapped angles, the figure uses color to show the size of the periodic phase shifts. The wave’s peak is illustrated on the positive side, while the bottom is depicted on the negative side. The size of the phase measures the amplitude of a wave in meters. The relative unwrapped phase image is distributed on a mesh based on antenna placements and split into a variety of rectangular forms. Each has a surface area of roughly 6 m^2^ (dx × dy), as shown in Figure 5.

The center frequency of 5.334999 MHz, the carrier bin of 5.351500 MHz, and a sampling rate of 100 kHz were the parameters of the dataset. The relative unwrapped phases were determined using LWA-stand SV’s number (antenna) 134 and LWA-1’s stand number (antenna) 10, both near the center of the antenna sites. The sample included cross-polarizations. A polarization of zero was used in the generation of the phase image.

For each time of data collection, a 2D Fourier transform over the space domain was performed to the relative phase image, *f*(*x*, *y*), to produce a complex Fourier image, F(u, *ν*), in the spatial frequency domain.

(2) $$F(u,\;\upsilon )=\int_{-\infty }^{\infty }\int_{-\infty }^{\infty }f(x,y)e^{-j2\pi (ux,\upsilon y)}\;\mathrm{dxdy}$$

where u and υ are spatial frequencies

(3) $$F(u,\upsilon )=F_{Re}(u,\;\upsilon )+jF_{Im}\;(u,\;\upsilon ),$$

The Fourier image’s amplitude is determined by

(4) $$\left|F(u,\upsilon )\right|\;=\sqrt{F_{Re}^{2}(u,\upsilon )+jF_{Im}^{2}(u,\upsilon )},$$

The Fourier image’s phase angle is calculated as

(5) $$\phi (u,\upsilon )={tan}^{-1}\left|\frac{F_{Im}(u,\upsilon )}{F_{Re}(u,\upsilon )}\right|,$$

The power of the wave is given by the amplitude square as

(6) $$P(u,\;\upsilon )={\left|F(u,\upsilon )\right|}^{2},$$

**Figure 6:**
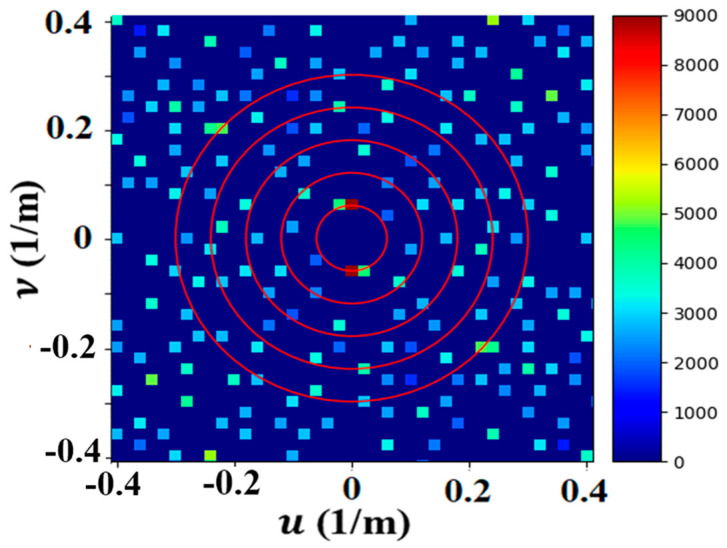
Fourier image shows symmetry in two dimensions, revealing many sets of waves existing in the phase image. The red circles in the plot show the frequency (0.06 cycles/m) in a series of harmonics.

The Fourier transform that produces the Fourier image from the phase image provides a convenient and effective method of separating the different sets of waves by their wavevectors and frequencies. The amplitudes of the Fourier peaks are the sizes of the phase shifts within the phase image. A representative Fourier image is shown in Figure 6, where each peak is paired with another peak of opposite wavevector and frequency symmetrically placed about the u and *υ*’s origin (0, 0).

## 3. Results

### 3.1. Analysis of LWA-SV and LWA-1 and Spatial Frequency Results

The experiment was conducted on 8 January 2021 at 20:30 UTC at the Santa Fe transmitter location (35.71144° N, 106.0084° W). The transmitted frequency was 5.3570 MHz, and the mode used was CW tone (continuous wave).

The experiment involved two receivers: LWA-SV (located at 34.348° N, 106.886° W) and LWA-1 (located at 34.069° N, 107.628° W). For the LWA-SV receiver, the date and time of the first frame recorded were 8 January 2021 and 20:29:20 UTC. The sample rate was 100,000 Hz, and the total recorded time was 1765.895 s. For the LWA-1 receiver, the date and time of the first frame recorded were 8 January 2021 and 18:00:00 UTC. The sample rate was also 100,000 Hz, and the total recorded time was 1731.281 s.

There were two places on the ionosphere being measured (Figure 1). They lay mid-way between Santa Fe and LWA-SV and between Santa Fe and LWA-1. Each place measured its own set of waves. Together, their stereographic projection revealed nicely the intersection points of the two sets of wavevectors having the same frequency of 0.06 cycles/m but different wavevectors [31]. Their intersection point represents the location of the source creating the waves as the ionospheric waves radiate outwards 360 degrees from the source. The local wave sources were previously determined to be Albuquerque and its nearby power generating stations [31].

**Figure 7:**
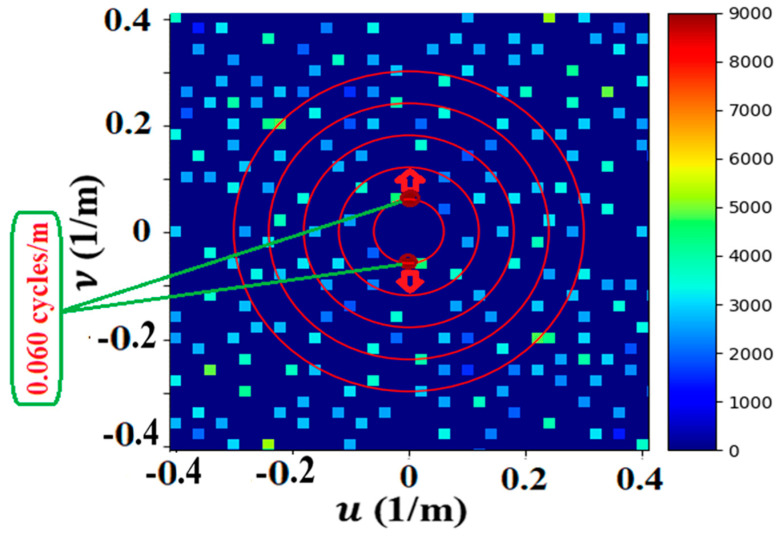
Fourier image of LWA-SV showing many peaks representing many sets of waves. The strongest set (red peaks) has 0.06 cycles/m with its wavevector (red arrows) pointing north–south. Greenish peaks have low power intensity, as shown in the scale bar. The red circles in the plot show the frequency (0.06 cycles/m) in a series of harmonics.

Figure 7 shows a Fourier image of the LWA-SV data revealing ~177 spatial frequency peaks. A set of strong peaks near the origin with a frequency of 0.06 cycles/m and wavelength of 16.667 m was selected to determine its power, represented by the two symmetric, yellow peaks in Figure 7.

#### 3.1.1. Model to Determine Wave Power

The power of the waves on the surface of the ionization layer was determined using a simple capacitor model. The analysis focused on the strongest set of waves, those represented by the two yellow Fourier peaks in Figure 7. These peaks were previously used to determine the origin of their disturbance at Albuquerque [31].

The ionosphere model employed two parallel conductive plates to represent both the Earth’s surface and the bottom surface of the ionosphere. The change in the distance between the plates, d, due to the amplitude of the waves, is the size of the measured phase shift in Figure 7. The surface area of the plates represents the imaged area of the antennas onto the surface of the ionosphere.

The change in capacitance, ∆C (farads), is given as

(7) $$∆C=\frac{{K\times \in }_{0}\times A}{∆d}$$

where *K* is the relative permittivity and equals 1 for vacuum and air; $\in_{0}$ is the permittivity of free space, $\in_{0}$ = 8.854 × 10^8^; *A* is the area illuminated on the ionosphere, $\frac{\pi \times (a\times b)}{4}$ (m^2^), where a and b are the elliptical coordinates (50 m and 55 m, respectively) determined from the size of the antennae array; and ∆d is the peak height, i.e., the change in distance between the plates (403 m) and equals the size of the phase shift of the wave. In terms of current and voltage, the change in capacitance is

(8) $$∆C=\frac{Q}{∆V}$$

where ∆*V* (volts) is the change in voltage across the capacitor. *Q* (coulombs) is the electron density in the ionosphere (3.5356 × $10^{11}$ ${(electrons/m}^{3})$ typically ranging from 10^10^ to 10^13^ electrons/m^3^, determined from the critical frequency for reflection/transmission of radio wave propagation within the ionosphere that occurs around 5 MHz, which was the carrier wave frequency used in this study. It should be noted, though, that the various ionosphere layers’ altitudes and their electron concentrations change according to the cyclical nature of solar radiation in every geographic area. Three factors taken into account in this study determined the ionosphere’s reflection. The first is the layer’s ion density. The second is the radio wave’s frequency, and the third is the angle at which the wave enters the ionosphere, close to 90 degrees from the vertically propagating wave.

**Figure 8:**
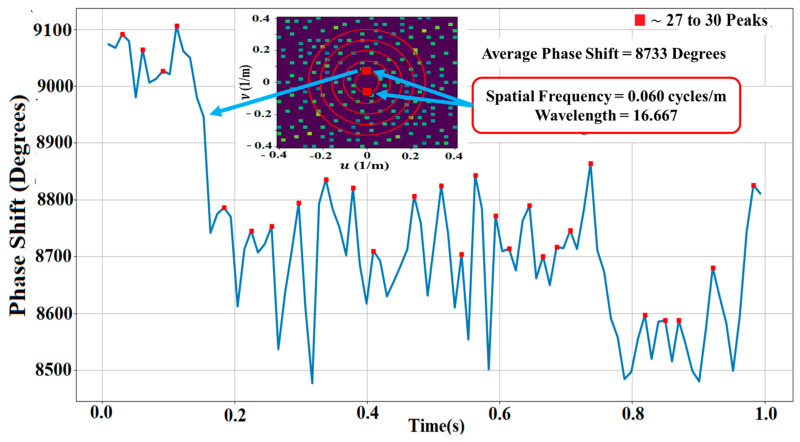
Phase shift measurements during one second of collection time having an average shift of 8707 degrees for the set of waves having 0.06 cycles/m.

The graph in Figure 8 shows the changes in the average size of the phase shift (degrees) in one second, which fluctuates over time. The size of the phase shift reached a low level of around 8500 degrees and a high level of around 9100 degrees, having an average phase shift of 8707 degrees.

The change in voltage of the capacitor was determined to be 1192 V. The current of the charge carried by the wave was determined to be 0.02468 A. The power of this set of waves was determined to be 14.7 W. This power increased to 15.3 W when translating it from the patch illuminated on the ionosphere to Albuquerque, a distance of 22 km, the source of the waves. A damping of 0.17/km was applied to the traveling wave determined from the reduction of the power of an ionospheric wave generated from the Nepali earthquake propagating to Taiwan and the Czech Republic [42].

The power density at Albuquerque was determined to be 0.018 W/m^2^ from the power, 15.3 W per area, i.e., (π × λ^2^), where λ was taken from the wavelength of the ionospheric wave, i.e., 16.7 m. In a similar manner, the total power density of all the sets of waves revealed in the Fourier image of Figure 7, for which there were 177, resulted in a power density of 0.1 W/m^2^ at the translated point of Albuquerque. This result is considered a low approximation since their sources of creation, which could be far and not local, were not determined.

##### Analysis of LWA-SV Spatial Frequency Results

Many local sources of power from generators within the state of New Mexico produce electromagnetic disturbances from their electric power stations and transmission lines. These disturbances were investigated for their power in context to the analysis of these results.

##### Local Power Generating Stations and Places of Measurements

**Figure 9:**
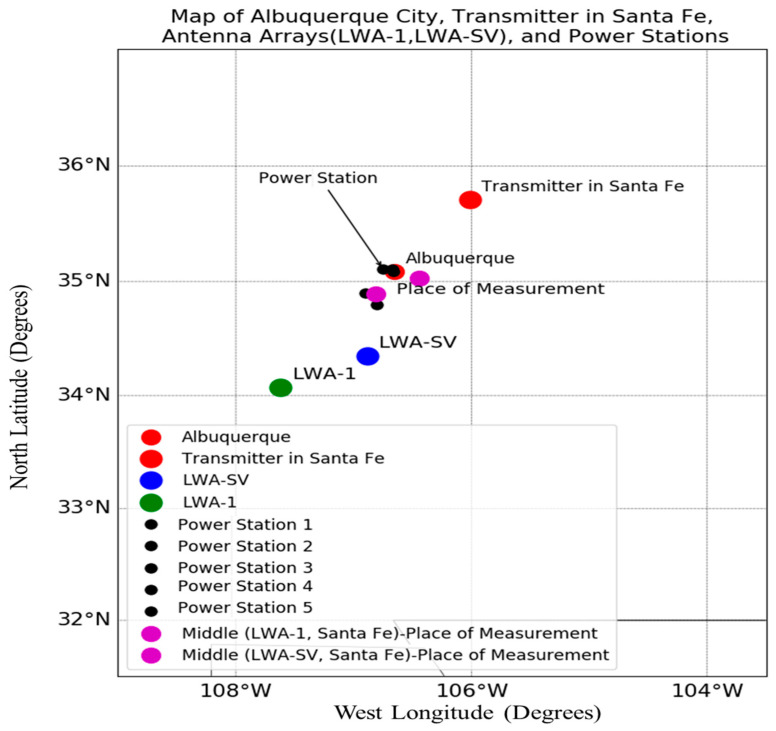
Map showing the power generating stations between the transmitter located in Santa Fe and LWA-SV and LWA-1.

Figure 9 displays the power generating stations around Albuquerque City and illustrates the expected measurement places between the transmitter in Santa Fe and the antenna arrays (LWA-SV and LWA-1).

The power, *I*, reaching the ionosphere generated by the power consumed from the local power generating stations around Albuquerque is given by

(9) $$I=\frac{P}{\pi \times r^{2}}$$

where *I* is the proportional power, *P* is the initial power, and *r* is the distance from the surface of the Earth to the ionosphere, determined to be around 78 km. Two power stations with high generating capacities of 156 × $10^{6}$ W each and three with low capacities for a total of 34.8 × $10^{6}$ W produces a total of ~347.4 MW. The proportional power transferred to the ionosphere above Albuquerque is 0.0018 W/m^2^.

## 4. Discussion of LWA-1 and LWA-2 Spatial Imaging Results

The receiving antenna array functioned as a camera, with each antenna representing one pixel in the phase image; hence, the 256 antennas in the array represented 256 pixels in the phase image, which had sufficient spatial resolution to show many sets of ionospheric waves. These sets of waves were analyzed using Fourier imaging, which separated the sets of waves by their frequencies and wavevectors. The strongest set of peaks having a spatial frequency of 0.06 cycles/m with a wavelength of 16.667 m was chosen for analysis of its power using a capacitor model, which resulted in this set of waves having 14.7 W. Translation from the patch on the ionosphere where the measurement occurred to Albuquerque, the source of the waves, the power increased to 15.3 W, producing a power density of 0.018 W/m^2^.

A calculation of the power density received by the ionosphere above Albuquerque produced from the total power of the local power generating stations was determined to be 0.018 W/m^2^, which was in agreement with the experimentally measured value. This agreement may imply that the power being generated by our power generators and consumed by industry and our homes is not lost but captured by the Earth’s ionosphere. 

The experimentally measured power density from all 177 sets of waves was approximated to be 0.1 W/m^2^, which still requires their sources to be determined and not provided in this study. In comparison to the solar constant of 1367 W/m^2^, a power density of 0.1 W/m^2^ is far less and may not significantly contribute to climate change even if it was linearly extrapolated to larger metropolitan cities 30 times larger than Albuquerque, although its importance for understanding climate change warrants further investigation.

## 5. Conclusions

The SDR Earth Imager enables the measurement of the power of ionospheric waves from phase images generated using radio waves transmitted terrestrially through the atmosphere, reflected from the ionosphere, and then detected terrestrially by an antenna array. The most powerful set of waves found within the phase image was measured to have a power density of 0.018 W/m^2^, which corresponded to the calculated power density of 0.018 W/m^2^ produced by the local power generating stations around Albuquerque, the place of generation of this set of ionospheric waves.

## Figures and Tables

**Table 1:** Transmitter signal and data set specifications were used during the LWA experiment.

**Santa Fe Transmitter (35.71144° N, 106.0084° W)**	
Date and Time	UT 8 January 2021, 20:29:30
Transmitted Frequency	5.3570 MHz
Mode -Send	CW Tone (Continuous Wave)
Receiver LWA-SV (34.348° N, 106.886° W)	
Center Frequency	5.33999 MHz
Polarization	Zero
Date and Time of First Frame	8 January 2021, 20:29:20
Sample Rate	100,000 Hz
Recorded Time	1765.895 s
Receiver LWA-1 (34.069° N, 107.628° W)	
Center Frequency	5.33999 MHz
Polarization	Zero
Date and Time of First Frame	8 January 2021, 18:00:00
Sample Rate:	100,000 Hz
Recorded Time	1731.281 s

## Data Availability

The dataset is available on Compute Canada Stores.

## References

[1] Ionospheric Detection of Natural Hazards—Astafyeva—2019—Reviews of Geophysics—Wiley Online Library. https://agupubs-onlinelibrary-wiley-com.ezproxy.library.uvic.ca/doi/full/10.1029/2019RG000668.

[2] Fedorov E.N., Mazur N.G., Pilipenko V.A. (2021). Electromagnetic Response of the Mid-Latitude Ionosphere to Power Transmission Lines. J. Geophys. Res. Space Phys..

[3] Liu Y., Zhou C., Tang Q., Chen G., Zhao Z. (2019). Geomagnetic Conjugate Observations of Ionospheric Disturbances in Response to a North Korean Underground Nuclear Explosion on 3 September 2017. Ann. Geophys..

[4] Rishbeth H. (2006). F-Region Links with the Lower Atmosphere?. J. Atmos. Sol.-Terr. Phys..

[5] Crowley G. (1985). Doppler Radar Studies of the Antarctic Ionosphere. Ph.D. Thesis.

[6] Crowley G., McCrea I.W. (1988). A Synoptic Study of TIDs Observed in the United Kingdom during the First WAGS Campaign, October 10–18, 1985. Radio Sci..

[7] Crowley G., Rodrigues F.S. (2012). Characteristics of Traveling Ionospheric Disturbances Observed by the TIDDBIT Sounder. Radio Sci..

[8] Crowley G., Jones T.B., Dudeney J.R. (1987). Comparison of Short Period TID Morphologies in Antarctica during Geomagnetically Quiet and Active Intervals. J. Atmos. Terr. Phys..

[9] Waldock J.A., Jones T.B. (1987). Source Regions of Medium Scale Travelling Ionospheric Disturbances Observed at Mid-Latitudes. J. Atmos. Terr. Phys..

[10] Liu J.-Y., Chen C.-H., Lin C.-H., Tsai H.-F., Chen C.-H., Kamogawa M. (2011). Ionospheric Disturbances Triggered by the 11 March 2011 M 9.0 Tohoku Earthquake. J. Geophys. Res. Space Phys..

[11] Tsugawa T., Saito A., Otsuka Y., Nishioka M., Maruyama T., Kato H., Nagatsuma T., Murata K.T. (2011). Ionospheric Disturbances Detected by GPS Total Electron Content Observation after the 2011 off the Pacific Coast of Tohoku Earthquake. Earth Planets Space.

[12] Nishioka M., Tsugawa T., Kubota M., Ishii M. (2013). Concentric Waves and Short-Period Oscillations Observed in the Ionosphere after the 2013 Moore EF5 Tornado. Geophys. Res. Lett..

[13] Crowley G., Azeem I. (2018). Chapter 23—Extreme Ionospheric Storms and Their Effects on GPS Systems. Extreme Events in Geospace.

[14] Hunsucker R.D., Hargreaves J.K. (2002). Radio Techniques for Probing the Ionosphere. The High-Latitude Ionosphere and Its Effects on Radio Propagation.

[15] Selman S. (2018). Traveling Ionospheric Disturbance (Tids) Characteristics Estimation from Gps-TEC over Ethiopian Longitudinal Sector. M.Sc Thesis.

[16] Pi X., Mannucci A.J., Lindqwister U.J., Ho C.M. (1997). Monitoring of Global Ionospheric Irregularities Using the Worldwide GPS Network. Geophys. Res. Lett..

[17] Dos Santos Prol F., Hernández-Pajares M., Tadeu de Assis Honorato Muella M., De Oliveira Camargo P. (2018). Tomographic Imaging of Ionospheric Plasma Bubbles Based on GNSS and Radio Occultation Measurements. Remote Sens..

[18] Sharif R., Tanyer S.G., Harrison S., Driessen P., Herring R. (2022). Monitoring Earth Using SDR Earth Imager. J. Atmos. Sol.-Terr. Phys..

[19] Crowley G., Azeem I., Reynolds A., Duly T.M., McBride P., Winkler C., Hunton D. (2016). Analysis of Traveling Ionospheric Disturbances (TIDs) in GPS TEC Launched by the 2011 Tohoku Earthquake. Radio Sci..

[20] Hunsucker R.D., Hargreaves J.K. (2002). Basic Principles of the Ionosphere. The High-Latitude Ionosphere and Its Effects on Radio Propagation.

[21] Hines C.O. (1960). INTERNAL ATMOSPHERIC GRAVITY WAVES AT IONOSPHERIC HEIGHTS. Can. J. Phys..

[22] Yeh K.C., Liu C.H. (1974). Acoustic-Gravity Waves in the Upper Atmosphere. Rev. Geophys. Space Phys..

[23] Francis S.H. (1975). Global Propagation of Atmospheric Gravity Waves: A Review. J. Atmos. Terr. Phys..

[24] Sun Y.-Y., Liu J.-Y., Lin C.-Y., Tsai H.-F., Chang L.C., Chen C.-Y., Chen C.-H. (2016). Ionospheric *F*
_2_ Region Perturbed by the 25 April 2015 Nepal Earthquake. J. Geophys. Res. Space Phys..

[25] Harrison R.G., Aplin K.L., Rycroft M.J. (2010). Atmospheric Electricity Coupling between Earthquake Regions and the Ionosphere. J. Atmos. Sol.-Terr. Phys..

[26] Zhang C., Ma Q. (2018). Influences of Radiation from Terrestrial Power Sources on the Ionosphere above China Based on Satellite Observation. IOP Conf. Ser. Earth Environ. Sci..

[27] Ando Y., Hayakawa M., Molchanov O.A. (2002). Theoretical Analysis on the Penetration of Power Line Harmonic Radiation into the Ionosphere. Radio Sci..

[28] Bullough K., Tatnall A.R.L., Denby M. (1976). Man-Made e.l.f./v.I.f. Emissions and the Radiation Belts. Nature.

[29] Molchanov O., Parrot M. (1995). PLHR Emissions Observed on Satellites. J. Atmos. Terr. Phys..

[30] Wu J., Guo Q., Yan X., Zhang C. (2019). Theoretical Analysis on Affecting Factors of Power Line Harmonic Radiation. IEEE Trans. Plasma Sci..

[31] Sharif R., Tanyer S.G., Harrison S., Junor W., Driessen P., Herring R. (2022). Locating Earth Disturbances Using the SDR Earth Imager. Remote Sens..

[32] Volland H. (1995). Handbook of Atmospheric Electrodynamics, Volume I.

[33] Parrot M., Zaslavski Y. (1996). Physical Mechanisms of Man-Made Influences on the Magnetosphere. Surv. Geophys..

[34] Helliwell R.A., Katsufrakis J.P., Bell T.F., Raghuram R. (1975). VLF Line Radiation in the Earth’s Magnetosphere and Its Association with Power System Radiation. J. Geophys. Res..

[35] Matthews J.P., Yearby K. (1981). Magnetospheric VLF Line Radiation Observed at Halley, Antarctica. Planet. Space Sci..

[36] Luette J.P., Park C.G., Helliwell R.A. (1977). Longitudinal Variations of Very-Low-Frequency Chorus Activity in the Magnetosphere: Evidence of Excitation by Electrical Power Transmission Lines. Geophys. Res. Lett..

[37] Park C.G., Miller T.R. (1979). Sunday Decreases in Magnetospheric VLF Wave Activity. J. Geophys. Res. Space Phys..

[38] Rothkaehl H., Izohkina N., Prutensky N., Pulinets S., Parrot M., Lizunov G., Blecki J., Stanislawska I. (2009). Ionospheric Disturbances Generated by Different Natural Processes and by Human Activity in Earth Plasma Environment. Ann. Geophys..

[39] Malins J.B., Obenberger K.S., Taylor G.B., Dowell J. (2019). Three-Dimensional Mapping of Lightning-Produced Ionospheric Reflections. Radio Sci..

[40] Varghese S.S., Obenberger K.S., Dowell J., Taylor G.B. (2019). Detection of a Low-Frequency Cosmic Radio Transient Using Two LWA Stations. Astrophys. J..

[41] Dowell J., Wood D., Stovall K., Ray P.S., Clarke T., Taylor G. (2012). The Long Wavelength Array Software Library. J. Astron. Instrum..

[42] Chum J., Liu J.-Y., Laštovička J., Fišer J., Mošna Z., Baše J., Sun Y.-Y. (2016). Ionospheric Signatures of the April 25, 2015 Nepal Earthquake and the Relative Role of Compression and Advection for Doppler Sounding of Infrasound in the Ionosphere. Earth Planets Space.

